# The efficacy of Ankaferd Blood Stopper^®^ in an experimental Asherman syndrome model created in rats

**DOI:** 10.4274/tjod.galenos.2018.21298

**Published:** 2019-03-27

**Authors:** Başak Büyük, Fatma Beyazıt

**Affiliations:** 1 Çanakkale Onsekiz Mart University Faculty of Medicine, Department of Histology and Embryology, Çanakkale, Turkey; 2 Çanakkale Onsekiz Mart University Faculty of Medicine, Department of Gynecology and Obstetrics, Çanakkale, Turkey

**Keywords:** Asherman syndrome, intrauterine synechiae, fibrosis, inflammation

## Abstract

**Objective:**

Asherman syndrome (AS) is a progressive disease involving menstrual disorders, recurrent pregnancy losses, and infertility developing as a result of partial or full blockade of the uterine cavity with adhesions. AS generally develops after trauma to the basal layer of the endometrium. In spite of a variety of methods such as adhesiolysis, inserting intrauterine devices, and administering high doses of estrogen, treatments remain insufficient. This study aimed to assess the effects of local intrauterine Ankaferd Blood Stopper (ABS) administration in inducing endometrial proliferation and building a normal endometrial layer in a rat model.

**Materials and Methods:**

AS was induced in 30 female Wistar albino rats. The rats were randomized into three groups:

Group 1: AS group

Group 2: AS + serum physiologic (SP) group

Group 3: AS + ABS group

AS model was induced in all animals. The uterine horns were harvested after 15 days of therapy and investigated for inflammation, fibrosis, and immunohistochemical (IHC) markers.

**Results:**

Compared with the other groups, fibrosis, and inflammation were significantly reduced in group 3 (chi-square, p=19.000, 0.001 and 26.365, <0.001, respectively). The IHC assessment showed that the tumor necrosis factor-α receptor levels were not different (Kruskal-Wallis H=0.091, p=0.995), but the interleukin (IL)-1β and IL-6 expression was reduced significantly in group 3 (H, p=18.706, <0.001, and 22.114, <0.001, respectively).

**Conclusion:**

The therapeutic effects of local administration of ABS in rats with AS model were demonstrated histopathologically and immunohistochemically. Based on these results, ABS administration in addition to the current treatments for AS may increase the treatment success and reduce the need for advanced treatment.

**PRECIS:** Ankaferd Blood Stopper can ameliorate intrauterine adhesions and inflammation on Asherman syndrome rat model.

## Introduction

Asherman syndrome (AS) is defined as the complete or partial obliteration of the uterine cavity with adhesions, resulting in amenorrhea or other menstrual aberrations, recurrent pregnancy loss, and infertility^(1)^. Although AS usually occurs after trauma to the basal layer of the endometrium due to dilatation and curettage, it can also be observed after a miscarriage, normal delivery, or medical abortion^(2)^.Uterine surgeries including myomectomy, metroplasty, and uterine septum resection are other potential causes of AS^(2,3)^.

Early detection and appropriate treatment by the removal of the adhesions could significantly improve the reproductive outcome of infertile women, promote the repair and regeneration of the destroyed endometrium, and resolve abnormal uterine bleeding complications. In this context, the actual management strategy of AS must be based on three key steps, including main treatment, re-adhesion prevention, and restoring normal endometrium^(2)^. Unfortunately, despite some therapeutic options, these management strategies still pose a serious challenge, and the overall prognosis (especially in moderate and severe cases) remains poor,^4^ indicating the need for new therapeutic approaches.

Ankaferd Blood Stopper (ABS) is a standardized herbal extract that has been used for hemostatic purposes in Anatolia for centuries^(5)^. ABS comprises standardized extracts of five medicinal herbs (i.e., Thymus vulgaris, Vitis vinifera, Glycyrrhiza glabra, Alpina officinarum, and Urtica dioica) that have special and unique effects on blood cells, endothelium, cellular proliferation, angiogenesis, and vascular dynamics^(6)^.The hemostatic properties of ABS provide a strict balance between thrombosis and hemorrhage by inducing a protein network formation with blood cells covering the primary and secondary hemostatic systems without disturbing individual coagulation factors. Besides its hemostatic properties, ABS also has considerable therapeutic benefits including the ability to act as an anti-inflammatory, anti-oxidant, and antineoplastic agent^(6)^.

At the cellular level, ABS has wound healing properties, which makes it a perfect candidate for mucosal disorders. Although there are no studies in the literature investigating the effect of ABS on the endometrial mucosa, experimental studies investigating the unique effects of ABS on the reduction and duration of chemotherapy-induced oral mucositis demonstrated favorable outcomes^(7)^.Moreover, ABS is shown to be effective in decreasing inflammatory response and accelerating wound healing in caustic esophageal injuries without any adverse effects on the gastrointestinal system mucosa^(8)^.

Using an experimental AS model, this study aimed to assess the effects of local intrauterine ABS administration in inducing endometrial proliferation and building a normal endometrial layer. Moreover, we aimed to determine whether local administration of ABS had an effect on the endometrial inflammatory response, which is associated with intrauterine adhesions (IUA).

## Materials and Methods

### Trial design

This study employed an experimental design with three randomization groups. The study was approved by the Çanakkale Onsekiz Mart University Experimental Animal Research Ethics Committee (approval number: 2016-04-05). Animal procedures were performed according to the “Guide for the Care and Use of Laboratory Animals” principles^(9)^.All steps of the study were conducted at the experimental research center of the university, open for supervision. Study reporting was performed in accordance with the CONSORT principles^(10)^.

### Participants

In this study, 30 female Wistar albino rats weighing 220-300 g were used. The rats were supplied by the university experimental research center. All rats were housed in pairs in appropriate cages in an animal room maintained at a standard humidity (45-50%) and temperature 22±2 °C with 12 hours light and 12 hours darkness, and were fed with standard food and water ad libitum.

### Randomization

The 30 rats were randomly divided into 3 groups. Randomization was performed by giving the rats sequential numbers and randomly assigning them to groups using a random numbers table. The groups were as follows:

Group 1: AS + no intervention group

Group 2: AS + serum physiologic (SP) administration group

**Figure 1:**
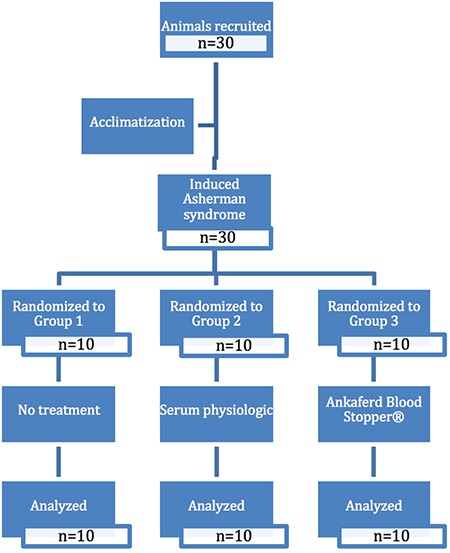
Randomization and participant flow

Group 3: AS + ABS administration group (Figure 1).

### Interventions

Vaginal smears were taken from all animals at the beginning of the study, and the menstrual cycles were synchronized.

All animals were anesthetized using 50 mg/kg ketamine hydrochloride (Ketalar®, Pfizer İlaçları Ltd. Şti. İstanbul, Turkey) and 10 mg/kg Xylazine (Alfazyne 2%, Ege Vet San. Tic. İzmir, Turkey) intraperitoneally. After achieving anesthesia, the vagina was entered using a 20-gauge branule. When the uterus bifurcation was reached, the branule was directed to the left to enter the left uterine horn. Here, 0.2 mL of trichloracetic acid (TCA) (IL-33, İstanbul İlaç San. Tic. AŞ, İstanbul, Turkey) was administered. After waiting 20 seconds, the branule was retracted, inducing the AS model.

Later, rats in all three groups were left for three menstrual cycles (15 days) to ensure the formation of the IUA. At the end of the 15^th^ day, rats in group 1 received no further intervention, whereas rats in group 2 were administered SP 2 mL/day into the left uterine horn using a 20-gauge branule via the transvaginal route under 2-3% isoflurane gas anesthesia. This intervention was repeated daily for 15 days.

At the end of the 15 days after TCA administration, rats in group 3 were administered ABS, which had a sterility statement given by Refik Saydam Hygiene Center, Ministry of Health of Turkey, at a dose of 2 mL/day into the left uterine horn using a 20 gauge branule via the transvaginal route under 2-3% isoflurane gas anesthesia for 15 days. At the end of the 15 day treatment period, a midline abdominal incision was applied to all rats under ketamine/xylazine anesthesia, followed by removal of the left uterine horn. The harvested tissues were stored in 10% neutral buffered formalin for histologic investigation.

### Outcomes

### Histopathologic evaluation

After the tissue processing protocol, the right and left uterine horns were embedded in paraffin blocks. Sections at a thickness of 4-5 microns were obtained from the paraffin blocks using a Leica RM 2125 RTS microtome and stained with routine hematoxylin-eosin (H&E) and Masson’s trichrome methods, followed by assessments with a Zeiss AxioScope A1 light microscope. Histopathologic assessments were completed and scored according to the method of Kilic et al^(1)^. for fibrosis and inflammation, which employs a scale ranging from 0 to 3 (0-no fibrosis, 1-minimal/loose fibrosis, 2-moderate fibrosis, and 3-dense fibrosis or 0-no inflammation, 1-presence of occasional lymphocytes and plasma cells, 2-presence of plasma cells, eosinophils, and neutrophils, and 3-presence of many inflammatory cells and micro abscesses).

### Immunohistochemical evaluation

IHC methods using anti-tumor necrosis factor (TNF) alpha receptor, anti- interleukin (IL)-1beta and anti-IL-6 primary antibodies were used to show inflammation in the uterus. After fixation in 10% neutral buffered formalin and routine histologic monitoring, the uterus tissues embedded in paraffin blocks were sliced into 4-micron sections using a Leica RM 2125 RTS microtome and mounted on adhesive slides. Antigen retrieval was applied to the sections, which were left at 65 °C for 1 hour and deparaffinized in xylene before passing through an alcohol series for rehydration. The antigen retrieval IHC method was performed at this stage.

Later, the sections were left in 10 mm EDTA (Thermo Scientific lot: Ax201208) for 20 minutes in a 200-watt microwave oven and then cooled at room temperature for 20 minutes. After cooling, each slide had sections outlined with a PAP pen. Then, 3% H_2_O_2_ (Thermo Scientific lot: HP31685) was dropped onto the sections and left in place for 15 minutes. Later, the samples were washed in phosphate-buffered saline with a pH of 7.4.

Lastly, all sections were incubated with anti-TNF-α (EMD Millipore Corporation, clone 13F9.1, lot: #Q2573230), anti-IL-6 (Santa Cruz Biotechnology, INC, sc-28343, lot: #I1316) and anti-IL-1β (Cell Signaling Technology, lot: #12242) primary antibodies, marked with AEC chromogen (Thermo Scientific lot: HA33805), and counterstained with Mayer’s hematoxylin before being covered. IHC assessment and scoring was completed according to the method of Jiang et al.,^11^ who used a formula combining both the staining intensity and the percentage of positively stained cells.

### Blinding

In this study, blinding was applied at the stage of the histopathologic investigation. Histopathologic assessments were performed by a single histologist, who had no knowledge about the groups.

### Statistical Analysis

Data analysis was performed using the SPSS software (version 20.0). Normal distribution of the data was checked using the Shapiro-Wilk test. Mean, standard deviation, median, maximum, and minimum values were used for descriptive data presentation. The Kruskal-Wallis test was used to compare numerical data between the groups. The chi-square test was used to compare groups for categorical data. P values of less than 0.05 were accepted as statistically significant.

## Results

### Participant flow

All recruited animals were followed up until analysis with no data loss (Figure 1).

### Recruitment

The study was conducted from June to November 2017. No problems were encountered necessitating cessation of the study.

### Outcomes and estimation

Both fibrosis and inflammation were less in group 3 compared with the other groups (chi-square, p=19.000, 0.001 and 26.365, <0.001, respectively). Although the majority of animals in groups 1 and 2 had levels 3 fibrosis and inflammation, there were no cases of fibrosis or inflammation to this extent in group 3 (Table 1, Graphic 1).

The mean IL-1 and IL-6 scores were significantly lower in group 3, but there were no significant differences between the groups concerning the TNF-α receptor scores (Table 2, Graphic 2).

**Figure 2:**
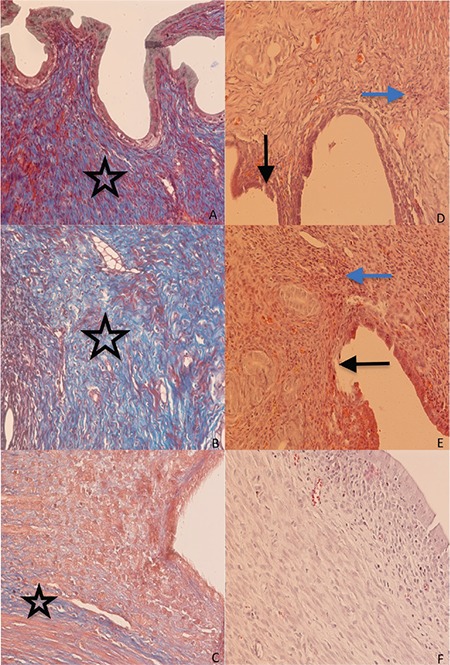
Microscopic comparison of fibrosis (A, B, C), and inflammation (D, E, F) in group 1 (no intervention), group 2 (serum physiologic), and group 3 (Ankaferd® Blood Stopper), respectively (x200 magnification). Group 1 and group 2 showed significantly greater fibrosis (stars) compared with group 3. Fibrosis level was decreased in group 3 with ABS treatment. Group 1 and group 2 showed significantly greater inflammatory cell infiltration (blue arrows) compared to group 3. Epithelial erosion areas that were seen in group 1 and group 2 (black arrows) were not seen in group 3. Group 1 and 2 showed significantly greater histologic damage including increased cellular inflammation, fibrosis, and erosion compared with the ABS-treated group

The histologic examination demonstrated that group 1 and group 2 showed significantly more fibrosis compared with group 3 (Figure 2). The fibrosis level was decreased in group 3, which received ABS. Also, group 1 and 2 showed significantly greater inflammatory cell infiltration compared with group 3 (Figure 2). Group 1 and 2 showed significantly greater histologic damage including increased cellular inflammation and fibrosis erosion compared with the ABS-treated group.

**Figure 3:**
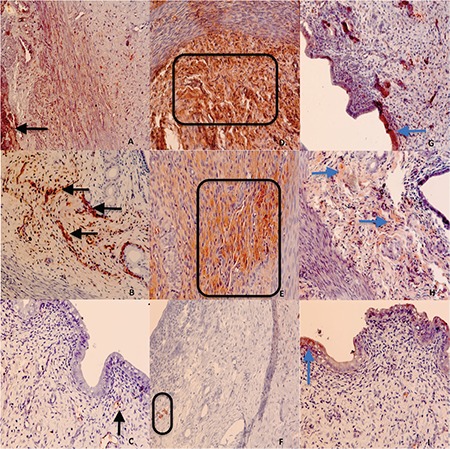
Immunohistochemical expression of IL-1 β in group 1 (A), group 2 (B) and group 3 (C); IL-6 in group 1 (D), group 2 (E), and group 3 (F); TNF-α receptor in group 1 (G), group 2 (H), and in group 3 (I) (x200 magnification). ABS treated group 3 demonstrated less prominent expression of IL-1 β (black arrows) and IL-6 (rectangles) compared with the other two groups (C and F, respectively). TNF-α receptor expression levels (blue arrows) were not significant between the groups (all figures taken x200 magnification)

As to the IHC examination, group 3 had a less-prominent expression of IL-1 β and IL-6 compared with the other groups (Figure 3).

## Discussion

In this study, we demonstrated that ABS administration in an AS rat model ameliorated uterine fibrosis and inflammation histopathologically. IL-1β and IL-6, which are acute inflammatory markers, were graded immunohistochemically, and it was shown that their levels were lower in the ABS-treated group. These findings show that local ABS administration can be helpful for treatment of AS.

AS is a health problem that is difficult to manage. Although not frequent perse, 13% of women treated for infertility are determined as having AS according to retrospective case series.^12^ Causes such as pregnancy, previous infections, missed abortus, uterine surgery, and curettage have been accused in the etiology^(13)^.

Histopathologically, the etiologic factors are thought to damage the stratum basalis layer, causing cyclic variations in the endometrium and IUA. The loss of the normal endometrial structure is accused as the underlying reason for infertility^(12)^. Once the basal layer of the endometrium is damaged, the stratum functionalis does not develop sufficiently during the menstrual cycle, and endometrial thickening does not occur. This large loss of the stratum basalis is called “endometrial sclerosis” and prepares the way for IUA^(2)^.

As to the medical literature, the treatment of AS generally appears to focus on opening the IUA and maintaining the opening in the uterine lumen^(2,12,14)^. In spite of surgically opening the IUA and administering medical treatment, adhesions recur in these patients. The resulting high infertility rates have revealed the need for the development of new treatment methods. Focusing on the endometrial stratum basalis in addition to the IUA during the treatment processes appears to be effective in ameliorating the inflammation. In this current study, ABS with previously proven hemostatic and anti-inflammatory effects were demonstrated to contribute to regulating the endometrial microenvironment, which may provide new aspects for the treatment of AS.

ABS is a medication containing standardized doses from five different plants. It has many proven effects on the hemostatic and immune system. In addition to antimicrobial, antineoplastic, antimutagenic, and antioxidant effects, it is also effective in wound healing^(5,15,16)^.The anti-inflammatory effect of ABS was shown in studies on cartilage tissue, gastric mucosa, pericardial tissue, and the liver^(17,18,19,20)^. A study on a caustic esophageal injury model, which demonstrated improved inflammation in the esophagus and reduced stricture formation, led to the consideration that the anti-inflammatory effects of ABS may be beneficial for the adhesions in the endometrium^(8)^. In our study, as expected, ABS was shown to ameliorate fibrosis, largely treat adhesions, and reduce endometrial inflammation.

Different studies have revealed the therapeutic effects of ABS in fibrosis and wound healing in different tissues such as pulmonary parenchyma, skin injuries, and intraabdominal adhesions^(21,22,23)^. In particular, the regression of fibrosis after administering ABS appears hope-promising for the management of IUA due to inflammation and fibrosis. These findings coincide with the regression in fibrosis when ABS was administered in the AS model.

TNF-α is an inflammatory cytokine released mainly from monocyte-macrophages. After TNF-α is released, it affects the TNF-α receptors^(24)^. In an acute gastric mucosal injury model, TNF-α values increased secondary to injury, which was shown to regress with ABS administration, suggesting that ABS improved inflammation and reduced TNF-α expression^(17)^.

In our study, the TNF-α receptor levels in the group administered ABS were not significantly different from the other groups. The TNF-α levels probably increased in response to the inflammatory process of the injury caused by TCA with an increase in TNF-α receptor expression, but the 15 day treatment may not suffice to reduce receptor expression. In fact, after ABS administration, the TNF-α levels reduced due to the anti-inflammatory effect as in all cytokines, with receptor expression reducing secondary to this decrease. Additionally, this study determined that IL-1β and IL-6 expression, increasing secondary to inflammation triggered by TCA administration to the uterus, reduced in IHC analyses. The reduction identified in the levels of the acute inflammatory markers of IL-1β and IL-6 clearly reveals the local anti-inflammatory effect of ABS.

Some researchers applied bone-marrow-derived stem cells (BMDSC) in the treatment of AS^(25,26,27,28,29)^. Autologous BMDSCs were administered with success to a patient receiving infertility treatment linked to IUA on 2011^(29)^. After this case report, a study was performed with six cases of grade 3 and 4 refractory AS on 2014^(28)^. BMDSCs were taken from iliac crest and implanted subendometrially via the transvaginal route. Patients were followed up at 3, 6, and 9 month intervals. Stem cell therapy was found efficient on AS and endometrial regeneration was obtained on patients in this study^(28)^. In 2016, a pilot study was performed with 16 patients whose ages ranged from 30 to 45 years^(27)^. In this study, researchers chose CD133+ BMDSCs, which have a neoangiogenetic effect, for stem cell therapy of refractory AS. CD133+ cells were isolated from peripheral blood and implanted subendometrially. At the end of this study, CD133+ BMDSC autologous cell therapy was found useful for treating patients with refractory AS and endometrial atrophy^(27)^. Apart from this, administration of adipose tissue-derived stem cells has been attempted as an alternative to BMDSCs and also here, successful results have been obtained^(1)^.

However, as stem cell administration requires multidisciplinary work and extensive infrastructure, its application is limited to centers with advanced facilities. In our study, we demonstrated successful administration of ABS in AS requiring limited resources. Administration of ABS does not require complex laboratory conditions, but can easily be applied in a simple examination environment. Especially in the first stages of AS treatment, local uterine administration of ABS may be attempted, reserving stem cell treatment choices for advanced cases with no response to the initial treatments. Pregnancy rates in rodents receiving stem cell treatment are promising in the long term^(25)^. On the other hand, studies of stem cell treatment for AS have shown endometrial thickening in later periods^(25,26,27,28,29)^.

### Study Limitations

This study has some limitations. First, we did not attempt to induce pregnancy after treatment. Another limitation was that the duration of our experiment was 15 days. If the treatment duration had been longer, perhaps the fall in TNF-α receptor levels would have reached statistically significant levels. However, in spite of this limitations, this is the first study in the literature to assess TNF-α receptor expression levels in an AS model. On the other hand, having included two control groups (one with no intervention and the other ‎SF administration) is a strength of this experiment.

### Generalizability

This study demonstrated the therapeutic effects of ABS on fibrosis and inflammation in Asherman syndrome. We think that the results are generalizable for further laboratory as well as clinical studies.

## Conclusion

In conclusion, the ameliorative effects of ABS on fibrosis and inflammation may contribute to the treatment process of IUA. The therapeutic effects of local administration of ABS in rats with AS model were revealed histopathologically and immunohistochemically. Based on these results, ABS administration in addition to the current treatments for AS may increase the treatment success and reduce the need for advanced treatments.

## Figures and Tables

**Table 1:**
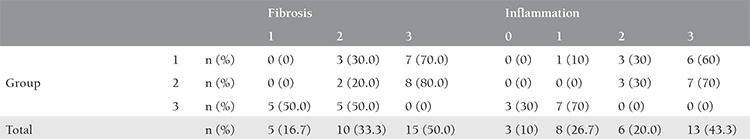
Comparison of fibrosis and inflammation levels between the groups

**Table 2:**
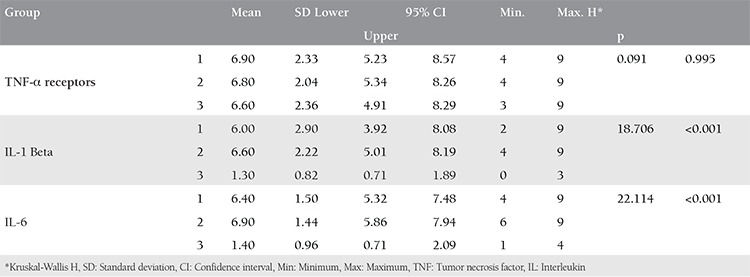
Comparison of mean immunohistochemical evaluation scores between the groups

**Graphic 1:**
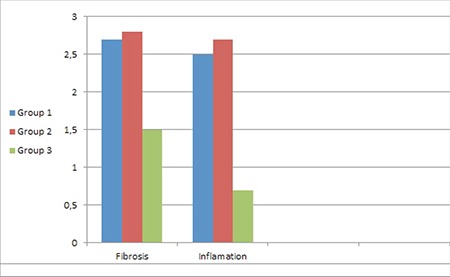
Comparison of the fibrosis and inflamation levels of all groups

**Graphic 2:**
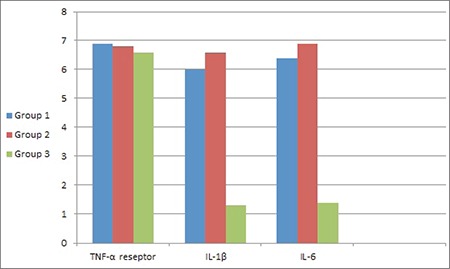
Comparison of the immunohistochemical results for TNF-α receptor, IL-1β and IL-6 levels of all groups
